# Drugs of Abuse Increase SGK1 Expression in the VTA

**DOI:** 10.51894/001c.143843

**Published:** 2025-09-30

**Authors:** Samantha Caico, Vedrana Bali, Sarah Simmons, Michelle Mazei-Robison

**Affiliations:** 1 College of Osteopathic Medicine Michigan State University

## 08

Activity within the mesolimbic dopaminergic (DA) circuitry in the brain is essential for motivated behavior and drugs of abuse can hijack this system by increasing DA signaling. Drug-induced cellular and molecular changes within the ventral tegmental area (VTA) can promote motivation to use drugs and drug-induced behavior. Our lab seeks to identify cellular and molecular changes in the VTA that promote drug responses. We found that repeated injections of morphine or cocaine increase the expression of serum– and glucocorticoid- inducible kinase 1 (SGK1) in the VTA using RNA sequencing. However, it was unclear whether similar regulation occurs with acute drug exposure and how persistent SGK1 expression changes are. To investigate this, we treated mice acutely with morphine or cocaine and isolated RNA from VTA for RT-PCR. We observed that VTA SGK1 gene expression is increased both 1 and 24 hours following both repeated and acute drug injections. We are currently exploring whether increased SGK1 expression persists during withdrawal and if it is differentially regulated by drug re-exposure. Given that the VTA is heterogenous structure containing multiple cell types besides DA neurons, we are determining which cell populations contribute to this drug-induced increase in SGK1 expression. We performed translating ribosome affinity purification studies to examine cell type-specific changes in SGK1 expression. Interestingly, we did not find an increase in SGK1 expression in either DA or GABA neuron pulldowns following morphine administration, suggesting that induction is occurring in alternative VTA cell populations or that SGK1 is not being actively translated. To assess whether SGK1 expression is occurring in specific neuronal populations or non-neuronal cells such as glia we are utilizing RNAScope analysis. Together, these studies will define how drugs of abuse alter expression of SGK1 in the VTA, a necessary first step to explore the role of this gene in drug behavior.

